# Grapevine Powdery Mildew: Fungicides for Its Management and Advances in Molecular Detection of Markers Associated with Resistance

**DOI:** 10.3390/microorganisms9071541

**Published:** 2021-07-20

**Authors:** Andrea Kunova, Cristina Pizzatti, Marco Saracchi, Matias Pasquali, Paolo Cortesi

**Affiliations:** Department of Food, Environmental and Nutritional Science (DeFENS), University of Milan, Via Celoria 2, 20133 Milan, Italy; cristina.pizzatti@unimi.it (C.P.); marco.saracchi@unimi.it (M.S.); matias.pasquali@unimi.it (M.P.); paolo.cortesi@unimi.it (P.C.)

**Keywords:** *Erysiphe necator*, fungicide mode of action, resistance, SNP, PCR

## Abstract

Grapevine powdery mildew is a principal fungal disease of grapevine worldwide. Even though it usually does not cause plant death directly, heavy infections can lead to extensive yield losses, and even low levels of the disease can negatively affect the quality of the wine. Therefore, intensive spraying programs are commonly applied to control the disease, which often leads to the emergence and spread of powdery mildew strains resistant to different fungicides. In this review, we describe major fungicide classes used for grapevine powdery mildew management and the most common single nucleotide mutations in target genes known to confer resistance to different classes of fungicides. We searched the current literature to review the development of novel molecular methods for quick detection and monitoring of resistance to commonly used single-site fungicides against *Erysiphe necator*. We analyze and compare the developed methods. From our investigation it became evident that this research topic has been strongly neglected and we hope that effective molecular methods will be developed also for resistance monitoring in biotroph pathogens.

## 1. Introduction

Grapevine powdery mildew is a major disease of cultivated and wild grapevine species worldwide causing substantial yield and economic losses [1]. It is caused by *Erysiphe necator* Schwein (previously *Uncinula necator* (Schwein.) Burrill; anamorph *Oidium tuckeri* Berk.), an obligate biotrophic fungus belonging to ascomycetes, family Erysiphaceae. The epiphytically growing mildew colonies can be observed as whitish, roughly circular spots, later assuming a typical powdery appearance due to abundant production of asexual conidia. The pathogen can infect all green tissues of the plant including leaves, shoots, flowers, and bunches, but the most economically important damage is due to flower and berry infections [1,2]. Early and severe infections can cause flower drop resulting in bunches with fewer berries, or as infected epidermal tissues of the berries stop growing, powdery mildew infection may cause berry splitting and facilitate the entrance of other grapevine pathogens. Moreover, apart from direct damage to the bunches, the infection can reduce photosynthesis and lower the sugar and anthocyanin content in the grape juice, leading to a lower content of total soluble solids and a less intense juice color and, on the other side, increase the acidity and the concentration of phenylacetic and acetic acid, which altogether causes inferior wine quality [2,3,4,5,6].

The vast majority of the cultivated grape varieties belonging to *Vitis vinifera* species have no genetic resistance to *Erysiphe necator* and result in being moderately to highly susceptible to grapevine powdery mildew [7,8,9]. Therefore, extensive fungicide programs are applied worldwide to keep the disease levels at a minimum. Fungicides are usually applied in a preventive manner, requiring multiple sprays per season, with 10–20 applications during the years favorable for epidemic development [10,11,12]. In fact, viticulture is a sector with one of the highest use of fungicides, with an average yearly application of 19.5 kg/ha of active ingredients [13]. Such extensive and repeated applications of fungicides increase the risk of resistance development in *E. necator* populations in different cultivation areas.

The situation is further complicated by the complex population structure of *E. necator*. High genetic diversity was observed in the Eastern North America, the presumed center of grapevine powdery mildew origin [14,15]. From here, it was most probably introduced in Europe and Australia, where two distinct genetic groups A and B (previously I and III) were repeatedly identified based on different molecular markers [16,17]. Some studies suggested that the group A is mostly clonal, associated with flag shoots, and can be recovered early in the growing season, while the group B is reproducing sexually, overwinters as chasmothecia and becomes dominant towards the end of the season [18,19]. Other studies, however, found high genetic variability in both genetic groups and approximately 1:1 mating-type ratios, not confirming the spatial and temporal diversification of the two groups [16,20]. However, higher sensitivity of the genetic group A was observed towards some fungicides, in particular triadimenol (DMI) and azoxystrobin (QoI), and it was hypothesized that this might be the reason for their precocious disappearance in vineyards [21,22].

In the following chapters, we briefly describe the different fungicide classes used for grapevine powdery mildew management and survey the presence of resistance to these classes. In case the molecular mechanisms of action are known, we describe the most common mutations responsible for the resistance in *E. necator* and other fungal plant pathogens, and finally, we discuss the advancements in the development of highly sensitive molecular methods for the detection of resistant strains and their implementation in practice.

## 2. Fungicides Used for Grapevine Powdery Mildew Management and Resistance in *E. necator*

A detailed overview of fungicides used against powdery mildews in different crop systems has been published recently [23]; here we will shortly describe those applied specifically for *E. necator* management. Currently, more than 20 fungicides belonging to different chemical classes are registered in the EU for powdery mildew management in vineyards (Table 1). Among them, the most numerous are fungicides targeting enzymes involved in electron transport within the mitochondrial membrane (FRAC classes 7, 11, and 29) and sterol biosynthesis (FRAC 3 and 5). Several classes, such as MBC-fungicides, SDHI, QoI, or SBI, encompass fungicides with a broad spectrum of activity used against different groups of pathogens, others such as hydroxy-(2-amino-) pyrimidines, aryl-phenyl-ketones, azanaphthalenes, and phenyl-acetamides target predominantly or are specific against powdery mildews.

### 2.1. Hydroxy-(2-Amino-)Pyrimidines

Hydroxypyrimidine fungicides belonging to FRAC 8 have specific activity against powdery mildews of different crops. They were introduced in the late 1960s, but nowadays, only bupirimate is registered in the EU against powdery mildews. Bupirimate is a systemic fungicide with translaminar mobility able to penetrate also woody tissues [24]. Moreover, its high vapor phase may contribute to good disease control properties [25]. Hydroxypyrimidines interfere at several stages of powdery mildew development, including germination, appressorium and haustorium formation, hyphal growth and sporulation, but the appressorium stage seems to be the most affected [26]. In particular, they act on the adenosine-deaminase enzyme involved in nucleic acid metabolism, catalyzing the deamination of adenosine. Synthesis of inosine and adenosine nucleotides is blocked by hydroxypyrimidines suggesting the inhibition of purine salvage pathway by these fungicides [24]. Shortly after their introduction in practice, resistance was observed in several powdery mildew species, including *Blumeria graminis* f. sp. *hordei* [27], and *Podosphaera xanthii* [28]. A gradual decrease in sensitivity of barley powdery mildew indicates a quantitative type of resistance [29]. It was hypothesized that resistance is not controlled by a single major gene but by the complex heritable system involving multiple genes with additive effect [30], however, the exact resistance mechanism is still unknown. Until now, no resistance has been described in *E. necator*. Due to pronounced resistance problems in powdery mildews of other principal crops such as cereals or cucurbits, this class of fungicides is nowadays of limited market importance [31].

### 2.2. MBC-Fungicides

Methyl benzimidazole carbamates (MBC) or benzimidazoles, classified as FRAC 1, were introduced in the late 1960s and represent an important class of fungicides. They were the first broad-spectrum systemic fungicides, also showing post-infection action, used mainly for foliar diseases or for seed dressing, and were characterized by low use rates [32,33]. Even though initially they were classified as having low toxicity to mammals and human, later negative effects on their reproduction and development were demonstrated [34,35], and in consequence, they were withdrawn from the EU agriculture, with the last active ingredient, thiophanate-methyl, having expired on 19 October 2020 [36]. The mode of action of MBC fungicides was elucidated in 1973 [37]; they inhibit microtubule assembly in diverse fungal species by binding to β-tubulin subunit and interfering with the polymerization, with subsequent disruption of the nuclear division, polarized growth, germ tube elongation, and mycelium growth. Only two years after their introduction in practice, field resistance was detected in multiple pathogens [38]. Currently, resistance to MBC was described in more than 100 fungal species, including different powdery mildew species [23,39]. A variety of single point mutations in the β-tubulin gene were described in laboratory-induced and field mutants, but the most important are E198A/G/K/Q and F200Y [39]. Interestingly, the mutations in the β-tubulin gene are not directly involved in binding with the benzimidazole fungicides but induce changes in the secondary structure of β-tubulin which prevent the binding of the fungicide [40]. Even though the MBC resistance was also detected in grapevine powdery mildew in the US [41,42], the underlying mutations were not investigated, presumably also due to lower importance of MBC fungicides in grapevine powdery mildew management.

**Table 1:** Fungicides used in grapevine powdery mildew management, their classification based on Fungicide Resistance Action Committee (FRAC) [43] and resistance in *E. necator*.

Mode of Action	Target Site and Code	Group Name	FRAC Code	Example A.I. ^a^	Resistance
**A**: Nucleic acid metabolism	**A2**: Adenosin-deaminase	Hydroxy-(2-amino-) pyrimidines	**8**	Bupirimate	ND ^c^
**B**: Cytoskeleton and motor protein	**B1**: β-tubulin assembly in mitosis	MBC-fungicides	**1**	Thiophanate-methyl ^b^	[41,42]
	**B6**: Actin/myosin/fimbrin function	Aryl-phenyl-ketones	**50**	Metrafenone, Pyriofenone	[11]
**C**: Respiration	**C2**: Complex II: succinate dehydrogenase	SDHI (Succinate-dehydrogenase inhibitors)	**7**	Boscalid, Fluopyram, Fluxa-pyroxad	[44,45,46]
	**C3**: Complex III: cytochrome bc1 (ubiquinol oxidase) at Qo site (*cyt b* gene)	QoI-fungicides (Quinone outside Inhibitors)	**11**	Azoxystrobin, Kresoxim-methyl, Pyraclostrobin, Trifloxystrobin	[47,48]
	**C5**: Uncouplers of oxidative phosphorylation		**29**	Meptyldinocap	ND
**E**: Signal transduction	E1: Signal transduction (mechanism unknown)	Azanaphthalenes	**13**	Proquinazid	[49]
**G**: Sterol biosynthesis in membranes	**G1**: C14-demethylase in sterol biosynthesis (*erg11/cyp51*)	DMI-fungicides SBI: Class I	**3**	Difeconazole, Fenbuconazole, Myclobutanil, Penconazole, Tebuconazole, Tetraconazole, Triadimefon	[50,51,52]
	**G2**: Δ14-reductase and Δ8→Δ7- isomerase in sterol biosynthesis (*erg24*, *erg2*)	Amines (morpholines) SBI: Class II	**5**	Spiroxamine	[48]
**U**: Unknown	Unknown	Phenyl-acetamide	**U06**	Cyflufenamid	ND
**M**: Multi-site	Multi-site contact activity	Inorganic	**M02**	Sulphur	ND

^a^ active ingredient; ^b^ registration expired on 19 October 2020 in the EU [36]; ^c^ not described yet. Letter codes of Mode of Action and Target site, and numerical FRAC codes are highlighted in bold.

### 2.3. Aryl-Phenyl-Ketones

Aryl-phenyl ketones (FRAC 50) are new-generation fungicides used almost exclusively for powdery mildew management. Currently, this group contains two fungicides; metrafenone was approved in the EU in 2007, followed by pyriofenone in 2014 [36]. Their exact mechanism of action has not been completely elucidated, but the studies on cereal powdery mildews suggested the disruption of the actin cytoskeleton and the interference with hyphal morphogenesis, polarized hyphal growth, and cell polarity [53,54]. Again, several years after their introduction, resistance was observed, first in *B. graminis* f. sp. *tritici* [55], followed by *E. necator* [11] and *P. xanthii* [56]. Interestingly, in grapevine powdery mildew, moderately- and highly-resistant isolates were observed; while both grew and sporulated at field dose of metrafenone, highly resistant strains were able to grow and sporulate similarly to control also at the concentration 1250 mg/L (10-times higher than the recommended field application) [11]. Resistant strains are nowadays distributed in different European countries at fluctuating frequencies and are not homogeneously distributed [45].

### 2.4. Succinate-Dehydrogenase Inhibitors

SDHI, or complex II inhibitors of mitochondrial respiration, belong to FRAC group 7. The first SDHI fungicides were discovered in the 1960s, however, they controlled only a narrow spectrum of plant pathogens [57]. In the 2000s, the second-generation, broad-spectrum SDHI were introduced, able to control a wide range of diseases. SDHI inhibit succinate dehydrogenase, an enzyme on the interface between the tricarboxylic acid cycle (TCA) and the mitochondrial respiratory chain, which couples the oxidation of succinate to fumarate in TCA with the reduction of ubiquinone to ubiquinol in the electron transfer chain. SDH is a complex, four-subunit enzyme and SDHI target the ubiquinone-binding pocket located at the interface between SdhB, SdhC, and SdhD subunits [44,58]. Various mutations in the three subunits involved in the formation of the ubiquinone-binding pocket have been described to be responsible for resistance to SDHI in different pathogens. In *E. necator*, a single isolate from a single vineyard in Italy with decreased sensitivity to fluxapyroxad was described during the European monitoring study in 2014 [45,59], a year later extensive resistance in French populations of grapevine powdery mildew to boscalid was described [44]. SDHI resistance was detected sporadically in different European countries, and in 2020, the monitoring detected only single sites in Hungary, France, and Germany [59]. In *E. necator*, two mutations in the SdhB subunit were identified: H242R/Y and I244V, and one in SdhC, G169D/S [44,45,59]. Of these, the substitutions in the SdhB subunit are homologous to known mutations from other plant pathogens, while SdhC-G169D/S substitution was described until now only in *E. necator* [45]. Interestingly, these mutations have a different impact on resistance to different SDHI fungicides. While H242R mutation confers resistance to boscalid (resistance factor RF > 100), it has no or little impact on the effectiveness of fluopyram and fluxapyroxad [44,45,46]. Instead, isolates carrying G169S mutation showed cross-resistance to both fluopyram and fluxapyroxad with RF > 10 [46].

### 2.5. Quinone Outside Inhibitors (Strobilurins)

Strobilurins, or quinone outside inhibitors (QoI, FRAC 11), represented a revolution in fungicide discovery and marketing, and they are still among the best-selling fungicides. They are derived from natural products, namely strobilurin A and oudemansin A produced by some basidiomycetes, which were further optimized to overcome the photo-liability and toxicity to mammals [60,61]. Azoxystrobin and kresoxim-methyl were put on the market in 1996, and currently, 18 QoI fungicides are registered for use in agriculture [43]. Their molecular mode of action has been studied in detail; they act within the inner mitochondrial membrane, in particular on complex III by binding to the quinol oxidation site (Qo) of cytochrome b [62]. This results in the disruption of electron transfer between cytochrome b and cytochrome c1, and in consequence, halts the ATP synthesis. Therefore, strobilurins are very effective in inhibiting energy-requiring processes of the pathogen development, in particular spore germination or zoospore mobility of a variety of plant pathogens [63]. Differences in sensitivity of *E. necator* to different strobilurin fungicides was observed, with the highest specific activity of pyraclostrobin (ED_50_ = 0.0044 mg/L), followed by azoxystrobin (ED_50_ = 0.013 mg/L), trifloxystrobin (ED_50_ = 0.015 mg/L), and relatively low activity of kresoxim-methyl [64]. Additionally, for QoI, resistance evolved very quickly in *E. necator* [47]. Among the three mutations known to confer QoI-resistance in different pathogens (F129L, G137R, and G143A), only the most common G143A was described in *E. necator*, and also in this case it is responsible for a high level of resistance with an RF > 1000 [65,66]. Nowadays, QoI-resistance is widespread in different parts of the world, including the US [66], Europe [67], or Australia [68,69]. Moreover, it seems that the G143A mutation does not confer a significant fitness penalty in *E. necator*, as resistance was described to persist for several years even in the absence of fungicide applications [65].

### 2.6. Uncouplers of Oxidative Phosphorylation

Uncouplers of oxidative phosphorylation are yet another class of fungicides targeting the complex pathway of mitochondrial respiration, classified as FRAC 29. In particular, this group of compounds increases the permeability of the membrane to small molecules and thus disrupts the proton gradient across the inner mitochondrial membrane necessary for the synthesis of ATP [60]. Dinocap, registered in the 1950s, was an important fungicide for powdery mildew management, discontinued due to its high eco-toxicity to non-target organisms and teratogenicity. Currently, meptyldinocap is the only fungicide in this class registered for powdery mildew management. It is the most potent isomer of dinocap, showing a much more favorable toxicological profile [70]. It was registered in 2007 and has both preventive and post-infectional activity inhibiting spore germination and mycelium growth and sporulation [70]. Despite the long use of this class, no resistance in *E. necator* and other powdery mildews has been described up until now, probably due to its nonspecific activity on the membrane permeability, and therefore, it remains a key fungicide in resistance management [71].

### 2.7. Azanaphthalenes

Azanaphthalenes, similarly to aryl-phenyl ketones, are a novel group of fungicides with specific activity against powdery mildews. They are classified as FRAC 13 group and currently only proquinazid, approved in 2010, is registered for use in agriculture. The registration of the other compound belonging to this class, quinoxyfen, was not renewed due to its high bioaccumulation and long persistence and was definitely withdrawn in March 2020 [36]. Proquinazid has strictly protectant activity with locally systemic and vapor phase redistribution and inhibits early stages of powdery mildew development such as spore germination and appressorium formation [72]. The exact mode of action of azanaphthalenes is not known, but the studies from barley powdery mildew suggest that they interfere with the appressorium development and perception of host-derived signals required for correct differentiation of the germinating spore [73,74]. Moreover, proquinazid induced the expression of *Arabidopsis thaliana* genes associated with resistance responses such as ethylene-mediated response pathway, phytoalexin biosynthesis, ROS generation, and induction of pathogenesis-related genes [49]. Gene-expression analysis of several genes involved in signal transduction evidenced differences in the mode of action between quinoxyfen and proquinazid, which indicates that, even though both interfere with signal transduction, their molecular targets might be different [23,74]. *Erysiphe necator* isolates collected in Europe, South Africa [75], and the US [76,77], with lower sensitivity to quinoxyfen were described, and a strong correlation (*r* = 0.874) between the sensitivity to proquinazid and quinoxyfen was observed [49]. The presence of such strains in vineyards does not seem to give problems in disease management, presumably due to a fitness penalty, as also observed in *B. graminis* f. sp. *hordei* [77,78].

### 2.8. Demethylation Inhibitors

Sterol biosynthesis inhibitors acting on the cytochrome P450 C14α-demethylase (CYP51) in the ergosterol biosynthetic pathway, classified as FRAC 3, are one of the most successful synthetic fungicide groups. The first DMI fungicides were registered in the 1970s, and currently there are seven compounds used in viticulture [43]. They are characterized by a broad spectrum of activity and are registered for use on diverse vegetables, crops, and fruit plants [61]. They usually do not affect conidia germination, but very efficiently inhibit the fungal growth by affecting the membrane integrity and functionality [4]. Their molecular mechanism of action has been studied in detail; DMIs bind to the heme iron of CYP51 with a nitrogen atom and inhibit the O_2_ binding and its transfer to lanosterol C14-methyl group, which is the main step in the lanosterol C14-demethylation process [79]. Despite their site-specific mode of action, the resistance to DMI has a quantitative character and is probably under polygenic control, and therefore, they still retain most of their activity [79]. Due to their extensive and repeated use, a shift in sensitivity to different DMI fungicides was observed in diverse countries, including the US and Chile [51,80], Europe and India [81], South Africa [52], and Australia [69]. Several mechanisms of resistance to DMI were described, such as single-point mutations in the *CYP51* gene, overexpression and/or copy-number variation of *CYP51*, and overexpression of efflux pumps [79]. A single-point mutation A495T leading to the amino acid substitution Y136F in the *CYP51* gene of *E. necator* was among the first described in 1997 and was characterized by the RF > 5 [80,81]. Additional non-synonymous mutations in the target gene have been described in other fungal pathogens, but only Y136F was detected in *E. necator* until now [82]. Moreover, a mutation A1119C leading to synonymous substitution in the *CYP51* sequence was associated with its overexpression and the azole resistance in *E. necator* [80]. This mutation does not change the amino acid sequence, and it was speculated that it could affect the mRNA stability, or it could be an unrelated marker in linkage disequilibrium with an unknown mutation, possibly in the promoter region, responsible for the overexpression. Interestingly, also a copy-number variation of *CYP51* together with the Y136F mutation was associated with fungicide resistance [4]. The authors hypothesized that an increase in copy number becomes advantageous when the *CYP51* gene is present in its fungicide-tolerant allelic form and can be adaptive in the development of resistance to DMI fungicides in *E. necator*. On the contrary, the overexpression of efflux pumps such as ATP-binding cassette (ABC) or major facilitator superfamily (MFS) transporters, known from other pathogens, has not been described in *E. necator* [79].

Apart from the detoxification mechanism due to the overexpression of ABC and MFS transporters and associated with multi-fungicide resistance [83,84], resistance to DMI is correlated with different mechanisms in a single target gene, *CYP51*, which makes the hypothesis of polygenic resistance quite controversial [85,86]. It was hypothesized, that apart from the major resistance gene, several additional genetic components might exist which, however, confer increased resistance to DMI only if the single major gene for resistance is already present, and their presence alone would not result in increased fungicide resistance [85]. Therefore, in-depth studies filling this knowledge gap are needed.

### 2.9. Amines (Morpholines)

Amines, or morpholines, belong to class II of sterol biosynthesis inhibitors (FRAC 5), their main target is Δ^14^ reductase, but they inhibit also squalene epoxidase, epoxysqualene cyclase, and sterol Δ^8^–Δ^7^ isomerase [87]. Currently, the only fungicide registered against grapevine powdery mildew is spiroxamine. Spiroxamine was registered in 1999 and is used especially on cereals and grapes. It has a protective, curative, and eradicative activity against powdery mildews and some rusts and spot fungi of cereals [87]. Morpholine fungicides show cross-resistance to their own members, but no cross-resistance to DMI or other fungicide classes has been observed [48]. Similarly to DMI, the resistance to amines is presumed to be multigenic and of quantitative type. The shift in sensitivity to triadimefon was observed in the Californian populations of *E. necator* [48], but regular monitoring in Europe reported a stable situation in the European countries with low resistance factors towards amines and only with small regional fluctuations close to the baseline [88]. The molecular basis of the reduced sensitivity in *E. necator* and other pathogens to amines has not yet been elucidated.

### 2.10. Cyflufenamid

Cyflufenamid, a more recent benzamidoxime fungicide registered in Japan in 2002 and the EU in 2005, has an excellent preventive and curative activity against powdery mildews in cereals and specialty crops, as well as brown rot on stone fruits [72,89]. Its exact mode of action is still unknown, and therefore it remains classified as a class FRAC U06 [43]. It is redistributed also in the vapor phase and has a good translaminar activity [72]. Early studies on *B. graminis* f. sp. *tritici* established that cyflufenamid does not inhibit the spore germination and appressorium formation, but strongly inhibits haustorium formation and further development of the fungus [90]. Also in the case of cyflufenamid, resistance developed only a few years after its introduction in practice, it was described in cucurbit powdery mildew in 2006 in Japan [91], followed by signalizations from Italy [92] and the US [93]. However, until now, no resistance was observed in grapevine powdery mildew.

To summarize, out of eleven FRAC classes currently employed for grapevine powdery mildew management, resistance in *E. necator* has been detected against fungicides belonging to seven classes (Table 1 and Table 2). However, also for bupirimate (FRAC 8) and cyflufenamid (FRAC U06), resistance has been observed in other powdery mildews such as *B. graminis* or *P. fusca*, and it is therefore possible that resistant *E. necator* will be identified shortly. Sulphur (FRAC M02) and meptyldinocap (FRAC 29), characterized by a non-specific and/or multi-site mode of action, remain the only fungicides where resistance has not been observed despite their prolonged and repeated use, and remain essential for resistance management strategies.

## 3. Molecular Detection Methods of Fungicide Resistance in *Erysiphe necator*

Resistance monitoring in grapevine powdery mildew remains challenging due to the fact that it is an obligate biotrophic pathogen. Currently, the most commonly used method for monitoring sensitivity to fungicides in *E. necator* and to detect resistance is the detached leaf or leaf-disk assay [71,94]. However, the use of these bioassays has several well-known drawbacks connected to working with obligate pathogens, such as constant availability of susceptible host plants, purification and difficult maintenance of individual strains of the pathogen, presence of sporulating colonies of the pathogen, etc. Detection and monitoring of resistance by conventional methods in most cases requires the isolation and purification of single-conidial isolates and evaluation of their response to one or several increasing concentrations of the fungicide, which is very laborious and time-consuming [95].

Therefore, the development of innovative and highly sensitive molecular methods is desirable for improved resistance management. They are, in general, faster, more sensitive, and more accurate, and often can be applied on a population scale and not only to single individuals. The detection of resistant individuals at low rates, before the resistance can be detected by traditional methods or before the failure of the fungicide treatments in the vineyard, could help in applying appropriate anti-resistance strategies and prolong the efficacy of the available fungicides.

However, the development and implementation of molecular methods requires knowledge of the genetic bases of fungicide resistance mechanisms in the fungal pathogen. Moreover, molecular monitoring can be more easily developed if only one or a few single-nucleotide mutations are associated with resistance; it becomes more challenging if multiple mutations in the target gene are known, or if different mechanisms contribute to resistance, e.g., SHDI or DMI. It is also important to stress that, on the contrary to bioassays which directly detect the resistant phenotype, i.e., the response of the pathogen to the applied fungicide in terms of conidia germination, growth, or sporulation, molecular methods detect the genetic markers—most often single-nucleotide polymorphisms (SNPs)—associated with resistance.

Despite resistance problems to many compounds, in-depth studies of the molecular mechanisms at the basis of resistance are often neglected, especially in obligate biotrophs such as *E. necator*. This in turn hinders the development of fast, accurate, and sensitive methods for resistance detection essential for supporting correct implementation of anti-resistance strategies. Indeed, mutations associated with resistance to fungicides in grapevine powdery mildew have been identified and studied in more detail only for SDHI, QoI, and DMI fungicides. Sequencing of target genes was performed to confirm known/discover new mutations [44,45,80,96,97]. Indeed, this approach led to the identification of the novel G169D/S mutation in the SdhC subunit of *E. necator* [45] and the synonymous mutation A1119C in the *CYP51* gene [80]. Until now, no specific assays for rapid detection of mutations in the SDH subunits have been developed. It would be important to understand the distribution, frequency, and overall importance of the novel mutation in SdhC in comparison with the mutations in the SdhB subunit.

qPCR-based methods have been developed in *E. necator* only for the most common Y136F mutation for the detection and monitoring of DMI resistance and G143A for the monitoring of strobilurin resistance (Table 3). In both cases, two different approaches were employed–allele-specific/amplification-refractory mutation system (ARMS) using wild-type (WT) and mutation (MUT) specific primers [81,96], or TaqMAN qPCR/ddPCR (digital droplet PCR) assays exploiting WT- and MUT- specific probes [65,97]. ARMS-qPCR utilize generally the cheaper SYBR Green technology, but two sets of PCRs are needed to assess the presence/absence of specific signals. Moreover, often, lower specificity is observed with non-specific detection of the fluorescence at high cycle levels, which might result in false-positive detection. On the other side, TaqMAN technology, even though considered more expensive, can detect both WT and MUT alleles in a single PCR, and is characterized with higher specificity.

Miles and coworkers [97] compared the specificity and ARMS-SYBR Green method developed by Baudoin et al. [96] with the TaqMAN qPCR and ddPCR for the detection of the G143A mutation in the cytochrome b gene of *E. necator*. They demonstrated higher specificity of the TaqMAN assay with the limit of detection (LOD) 0.05 ng/mL compared to the ARMS-SYBR Green assay. However, the qPCR assay was able to detect the WT or MUT allele in mixed samples only when the allele concentration in the mixed sample reached 10%, while ddPCR assay was able to detect either allele if it was present in 1% of the sample [97].

Different qPCR assays were developed also for the marker A495T in the *CYP51* gene coding for Y136F mutation associated with DMI resistance, exploiting both SYBR Green [98,99] and TaqMAN technology [65,100]. Employing the SYBR Green technology, LOQ (limit of quantification) of the mutant allele in the mixture was 2.85% [98], and for the TaqMAN assay the authors did not provide the data on the assay development such as the sensitivity and specificity of the assay.

As mentioned before, different molecular mechanisms are responsible for DMI resistance and, apart from assays for the detection of A495T marker, development of quantitative methods to detect *CYP51* overexpression and copy number would be needed. The combination of such methods would contribute to elucidating the impact of each mechanism on field resistance development and maintenance.

**Table 3:** Molecular methods developed for the detection of the SNP markers of the fungicide resistance in *Erysiphe necator*.

Target Gene	Mutation	Molecular Method	Primer Description	Primer Sequence (5′->3′)	Ref
Succinate dehydrogenase	SdhB-H242R/Y,I244V	PCR-sequencing	Forward	AGACGAAGCTGTAGAGAGGGT	[44]
			Reverse	GCTGGAGAAAAACGCCTTTCAA	
	SdhC-G169D/S	Pyrosequencing	Forward	Biotin-ACATGGGAAAGGCTTTTACAAAT	[45]
			Reverse	ACCAAAGCTACCAAAGCTAATGC	
			Sequencing primer	ATCCAACTACCATCCAG	
Cytochrome b	G143A	ARMS ^a^-qPCR	Forward-WT ^b^	TACGGGCAGATGAGCCTATGCG**G**	[65,96]
			Forward-Mut ^c^	TACGGGCAGATGAGCCTATGCG**C**	
			Reverse	ACCTACTTAAAGCTTTAGAAGTTTCC	
		TaqMAN-qPCR	Forward	CGCTACAGACTGGGTCACTG	[97]
			Reverse	AGTCTCTTAGGGCCCCCATT	
			Probe-WT	AGCCTATGGG**G**TGCAACCGT	
			Probe-Mut	AGCCTATGGG**C**TGCAACCGT	
C14-demethylase	Y136F	PCR-Sequencing	Forward	TCATCTCTTTTCCCAGCCTATC	[101]
			Reverse-Mut	GTATTGAGGCGGGTAAATCG	
		Allele-specific PCR	Forward	ATGTACATTGCTGACATTTTGTCGG	[81]
			Reverse-Mut	AATTTGGACAATCA**A**	
		Allele-specific qPCR	Forward	TGGGAAGTTAAAAGATGTCAACG	[98]
			Reverse-Mut	TGAGTTTGGAATTTGGACAATCA**A**	
		Allele-specific qPCR	Forward	CGCCGAAGAGATTTACACTA	[99]
			Reverse-Mut	TGAGTTTGGAATTTGGACAATCA**A**	
		TaqMAN-qPCR	Forward	ACTAATTTAACAACTCCGGTCTTTGGA	[65,100]
			Reverse	ACTCGACCATTTACGGACCTTTTT	
			Probe-WT	VIC-TTGGACAATCA**A**ATACAAC	
			Probe-Mut	FAM-TTTGGACAATCA**T**ATACAAC-3	
	A1119C	PCR-Sequencing	Forward	TTCTGATGGCTGGACAACAC	[80]
			Reverse	AACCCTAACACCTGCCATAAA	

Different methods used for the detection of single nucleotide mutations in the target genes and primers used are described. ^a^ Amplification Refractory Mutation System, ^b^ Wild type, ^c^ Mutant (resistant).

## 4. Conclusions

The resistance of grapevine powdery mildew to different fungicide classes is an extensive problem common to all grapevine growing regions, but the development of novel sensitive techniques that could be routinely used for early detection of resistant isolates and improve resistance management is still slow. This is further aggravated by the fact that *E. necator* is an obligate biotroph, which makes the research even more challenging. In this review, we summarized the recent advances in the development of molecular methods for the detection of resistance in *E. necator*, and from our investigation it is clear that this research topic has been strongly neglected. We hope that this review will contribute to making the investigation of resistance mechanisms in biotroph pathogens such as *E. necator* more attractive.

## Figures and Tables

**Table 2:** Molecular mechanisms of resistance to fungicides applied in grapevine powdery mildew management. Target gene and single-nucleotide mutations described in various fungal pathogens and grapevine powdery mildew.

FRAC Code	Group Name	Target Gene	Mutations Associated with Resistance in	
			Various Fungal Pathogens	*E. necator*
**8**	Hydroxy-(2-amino-) pyrimidines	adenosin-deaminase	unknown ^a^	not detected ^b^
**1**	MBC-fungicides	β-tubulin	E198A/G/K/Q, F200Y	not investigated
**50**	Aryl-phenyl-ketones	unknown	unknown	unknown
**7**	SDHI (Succinate-dehydrogenase inhibitors)	succinate dehydrogenase	various mutations mostly in SdhB gene	SdhB H242R/Y, I244V, SdhC G169D/S
**11**	QoI-fungicides (Quinone outside Inhibitors)	cytochrome b	F129L, G137R, G143A/S, other mechanisms	G143A
**29**	Uncouplers of oxidative phosphorylation	unknown	not detected	not detected
**13**	Azanaphthalenes	unknown	unknown	unknown
**3**	DMI-fungicides SBI: Class I	C14-demethylase	V136A, Y137F, A379G, other mechanisms	Y136F, A1119C
**5**	Amines (morpholines) SBI: Class II	Δ^14^-reductase, Δ^8^→Δ^7^- isomerase	unknown	unknown
**U06**	Phenyl-acetamide	unknown	unknown	not detected
**M02**	Inorganic	multi-site	not detected	not detected

^a^ resistance detected but the molecular mechanism unknown; ^b^ resistance not detected. Numerical FRAC codes are highlighted in bold.
